# The Economic Utility of an Alzheimer's Disease Blood Biomarker Test in the Evaluation of Cognitively Impaired Patients

**DOI:** 10.1002/alz70860_106232

**Published:** 2025-12-23

**Authors:** William J Canestro, Mark Monane, Randall J. Bateman, David M. Holtzman, Joel B. Braunstein

**Affiliations:** ^1^ University of Washington Institute for Protein Design, Seattle, WA, USA; ^2^ C2N Diagnostics, LLC, Saint Louis, MO, USA; ^3^ Washington University School of Medicine, St. Louis, MO, USA; ^4^ C2N Diagnostics, LLC, St. Louis, MO, USA

## Abstract

**Background:**

Blood biomarkers (BBMs) represent an acceptable, accessible, equitable, and scalable alternative to amyloid positron emission tomography (PET) or cerebrospinal fluid (CSF) biomarkers to help establish an Alzheimer's disease diagnosis. The PrecivityAD2 test (C2N Diagnostics, St. Louis, MO) is a multi‐analyte assay with algorithmic analysis (MAAA) BBM test that detects presence of brain amyloid plaques with high diagnostic accuracy among patients undergoing evaluation for cognitive impairment. While the clinical validity of the PrecivityAD2 test has been established, its budgetary impact has not been well described.

**Method:**

Our budget impact model, based on a decision tree that considered a one million (1MM) US health plan population, evaluated the effects of PrecivityAD2 test adoption as a first evaluation at the neurologist's office versus the first current standard use of PET or CSF testing in patients with cognitive impairment. We used estimates of real‐world adoption, adherence, and drop‐out measures drawn from clinical expert opinion as well as clinical utility study data, rather than idealized scenarios, to simulate clinical practice more closely.

**Result:**

A clinical pathway using the PrecivityAD2 test early in the evaluation process had improved sensitivity (90.1% vs 93.5%) and reduced specificity (92.3% vs 84.8%) as compared to usual care utilizing PET or CSF biomarkers as first‐line biomarker testing. Net savings from PrecivityAD2 test use in the diagnostic work‐up was $10.9MM or $0.91 per member per month (PMPM) in this 1MM lives model, representing a 26.2% reduction in total costs for AD evaluation. Among diagnosed cases in the model, PrecivityAD2 testing resulted in cost savings of $2,753 and $3,578 per case identified at 40% and 100% test penetration, respectively. Savings were driven by reductions in utilization of PET and CSF testing. The potential for economic impact is significant: at a 40% adoption rate, savings to the US healthcare system could exceed $3.5B dollars.

**Conclusion:**

In a budget impact model, PrecivityAD2 testing prior to usual care brain amyloid detection methods in patients undergoing evaluation for cognitive impairment provides cost savings and reduces the predicted burden on both patients and payers, while maintaining a high quality of care.

